# Correction: Ecological niche modelling of two water-dependant birds informs the conservation needs of riverine ecosystems outside protected area network in the Eastern Himalaya, India

**DOI:** 10.1371/journal.pone.0303884

**Published:** 2024-05-13

**Authors:** Roshan Tamang, Vallanattu James Jins, Sailendra Dewan, Shivaji Chaudhry, Seema Rawat, Bhoj Kumar Acharya

In Table 1, the percentage value in column Blue Whistling Thrush of S.L No.1, Elevation Zone: 0–1000 is incorrect. The correct percentage value is 25.50%. Please see the correct Table 1 here.

**Table 1:** High suitability areas of both bird species in each elevation zone in the Sikkim Himalaya. The value within the bracket shows the percentage with respect to total suitable area.

**Sl.**	**Elevation zone (m)**	**Suitable habitat area (km** ^**2**^ **)**	
		**Blue Whistling Thrush**	**White-capped Water Redstart**
1	0–1000	399.16 (25.50%)	393.71 (23.19%)
2	1000–2000	838.78 (53.58%)	818.55 (48.21%)
3	2000–3000	190.63 (12.18%)	281.67 (16.59%)
4	3000–4000	111.27 (7.11%)	117.49 (6.92%)
5	4000–5000	17.12 (1.09%)	52.13 (3.07%)
6	5000–6000	8.56 (0.55%)	34.24 (2.02%)
**Total**		1565.51	1697.78
