# Supplementary material for: Ecological niche modelling of two water-dependant birds informs the conservation needs of riverine ecosystems outside protected area network in the Eastern Himalaya, India
Source: PLoS One. 2023 Nov 9;18(11):e0294056. doi: 10.1371/journal.pone.0294056 (PMC10635460; doi:10.1371/journal.pone.0294056)
Supplement: S1 Table — (DOC) [file pone.0294056.s001.doc]

**S1 Table.** Details of sighting records of Blue Whistling Thrush in different parts of Sikkim Himalaya.

| **Elevation** | **Forest type** | **Location** | **District** | **Latitude** | **Longitude** |
| --- | --- | --- | --- | --- | --- |
| 300 | TrSDF | Bardang | East | 27.2017 | 88.4817 |
| 450 | TrSDF | Dalep | South | 27.2467 | 88.4533 |
| 510 | TrSDF | Lingmoo | South | 27.5100 | 88.5081 |
| 600 | TrSDF | Pabong | South | 27.2383 | 88.4733 |
| 640 | TrSDF | Sumbuk R.F. | South | 27.1069 | 88.3667 |
| 700 | FAS | Lower Bering | East | 27.7100 | 88.6594 |
| 750 | TrSDF | Namphing | South | 27.2517 | 88.4433 |
| 750 | MOAS | Lingee Suntaley | South | 27.3733 | 88.4828 |
| 750 | TrSDF | Lingmoo | South | 27.5000 | 88.5089 |
| 800 | FAS | Upper Bering | East | 27.2133 | 88.6542 |
| 850 | MOAS | Lingee Karjee | South | 27.3731 | 88.4747 |
| 850 | TrSDF | Tareythang | East | 27.2203 | 88.6444 |
| 850 | TrSDF | Lingmoo | South | 27.5267 | 88.5528 |
| 900 | TrSDF | Sankalang | North | 27.4883 | 88.5100 |
| 980 | FAS | Sumbuk Kamarey | South | 27.1200 | 88.3892 |
| 1000 | MOAS | Lower Pendam | East | 27.2022 | 88.5225 |
| 1030 | FAS | Sumbuk | South | 27.1136 | 88.3778 |
| 1050 | TrMBF | Sankalang Toong | North | 27.4900 | 88.5117 |
| 1100 | MOAS | Sang Sakim | East | 27.2469 | 88.5000 |
| 1200 | TrMBF | Barphok | North | 27.4917 | 88.5033 |
| 1200 | LCAS | Luing | East | 27.3594 | 88.5850 |
| 1250 | MOAS | Sang Bhirkuna | East | 27.2650 | 88.4961 |
| 1280 | LCAS | Lower Luing | East | 27.3539 | 88.5944 |
| 1350 | TrMBF | Toong | North | 27.5517 | 88.6417 |
| 1450 | FAS | Gumpa Dara, Payong | South | 27.3692 | 88.4581 |
| 1500 | TrMBF | Theeng | North | 27.5700 | 88.6533 |
| 1500 | LCAS | Khamdong | East | 27.2889 | 88.4661 |
| 1540 | MOAS | Upper Pendam | East | 27.2167 | 88.5247 |
| 1550 | FAS | Green Village, Payong | South | 27.3675 | 88.4517 |
| 1597 | TrMBF | Kaw | South | 27.3639 | 88.4508 |
| 1610 | LCAS | Pantharey, Payong | South | 27.3886 | 88.4428 |
| 1650 | TrMBF | Chungthang | North | 27.6033 | 88.6433 |
| 1750 | LCAS | Simkharka, Payong | South | 27.3714 | 88.4400 |
| 1800 | TBF | Bop | North | 27.6183 | 88.6683 |
| 1800 | TrMBF | Tumin R.F. | East | 27.2950 | 88.4775 |
| 1850 | TBF | Sumin R.F. | East | 27.2236 | 88.5494 |
| 1900 | TBF | Menshithang | North | 27.6267 | 88.6150 |
| 1970 | TBF | Bulbuley | East | 27.3422 | 88.6317 |
| 2000 | LCAS | Khamdong | East | 27.2969 | 88.4744 |
| 2000 | TBF | Maenam WLS | South | 27.3714 | 88.4342 |
| 2010 | TBF | Rabdantse | West | 27.3011 | 88.2539 |
| 2150 | TBF | Khedum | North | 27.6283 | 88.7033 |
| 2400 | TBF | Rabum | North | 27.6633 | 88.6050 |
| 2650 | TBF | Bitchu | North | 27.6583 | 88.7283 |
| 2850 | TCF | Lachung | North | 27.6850 | 88.7567 |
| 3050 | TCF | Phuni | North | 27.7533 | 88.7300 |
| 3250 | TCF | Shingba | North | 27.7800 | 88.7083 |
| 3450 | TCF | Yumthang | North | 27.8067 | 88.7117 |
| 3650 | TCF | Yumthang | North | 27.8217 | 88.7083 |
| 4000 | SAP | Shivmandir | North | 27.8567 | 88.6933 |
| 4350 | SAP | Yumesamdong (South) | North | 27.8700 | 88.6950 |
| 4700 | ALP | Yumesamdong (North) | North | 27.9133 | 88.6983 |

TrSDF- Tropical Semi-deciduous forests, TrMBF- Tropical moist and broad-leaved forests, TBF- Temperate broad-leaved forests, TCF- Temperate coniferous forests, SAP- Sub-alpine vegetation, AP- Alpine zone, FAS- Farm-based agroforestry system, MOAS- Mandarin orange-based agroforestry system, LCAS-Large cardamom-based agroforestry system.
