# Supplementary material for: Ecological niche modelling of two water-dependant birds informs the conservation needs of riverine ecosystems outside protected area network in the Eastern Himalaya, India
Source: PLoS One. 2023 Nov 9;18(11):e0294056. doi: 10.1371/journal.pone.0294056 (PMC10635460; doi:10.1371/journal.pone.0294056)
Supplement: S2 Table — (*) Data obtained from others. (DOC) [file pone.0294056.s002.doc]

**S2 Table.** Details of sighting records of White-capped water redstart obtained in different parts of Sikkim. (*) Data obtained from others.

| **Elevation** | **Habitat type** | **Location** | **District** | **Latitude** | **Longitude** |
| --- | --- | --- | --- | --- | --- |
| 356 | TrSDF | Singtam | East | 27.23 | 88.48 |
| 364 | TrSDF | Tshalamthang | South* | 27.20 | 88.48 |
| 391 | TrSDF | Tarku | South* | 27.25 | 88.44 |
| 570 | TrSDF | Dikchu | North | 27.40 | 88.51 |
| 621 | TrSDF | Lum | South* | 27.37 | 88.47 |
| 627 | MOAS | Gelling | West | 27.16 | 88.26 |
| 641 | FAS | Linzey | East | 27.23 | 88.53 |
| 715 | TBF | Barfok | North* | 27.49 | 88.52 |
| 774 | FAS | Khamdong | East | 27.26 | 88.48 |
| 930 | MOAS | Yangtey | West | 27.30 | 88.27 |
| 1110 | MOAS | Thekabung | East* | 27.23 | 88.64 |
| 1220 | LCAS | Upper Paiyong | East* | 27.36 | 88.45 |
| 1271 | LCAS | Dentam | West | 27.26 | 88.13 |
| 1321 | LCAS | Sombaria | West | 27.13 | 88.15 |
| 1326 | FAS | Tung | North* | 27.55 | 88.64 |
| 1360 | LCAS | Deythang | West | 27.21 | 88.22 |
| 1378 | LCAS | Luing | East* | 27.35 | 88.59 |
| 1417 | TrMBF | Sadam | South | 27.13 | 88.41 |
| 1451 | TrMBF | Paegum | North* | 27.57 | 88.65 |
| 1511 | FAS | Parbing | East* | 27.35 | 88.57 |
| 1533 | FAS | Sokpay | East* | 27.39 | 88.43 |
| 1589 | TrMBF | Chungthang | North* | 27.60 | 88.64 |
| 1654 | LCAS | Soreng | West | 27.17 | 88.19 |
| 1702 | TrMBF | Sangkhu | West* | 27.23 | 88.13 |
| 1798 | TrMBF | Khecheopalri | West | 27.34 | 88.18 |
| 1806 | LCAS | Barakheley | West | 27.18 | 88.20 |
| 1820 | LCAS | Sribadam | West | 27.21 | 88.20 |
| 1902 | TBF | Lingi | East* | 27.37 | 88.43 |
| 1909 | LCAS | Gumpadara | West | 27.17 | 88.18 |
| 2202 | TBF | Khedum | North* | 27.62 | 88.70 |
| 2281 | TBF | Gorkhey | West | 27.18 | 88.07 |
| 2311 | TBF | Bhareng | West | 27.17 | 88.09 |
| 2562 | TBF | Latong | North* | 27.66 | 88.62 |
| 3383 | TCF | Dambung | North* | 27.75 | 88.73 |
| 3556 | TCF | Lachung | North* | 27.78 | 88.71 |
| 3600 | TCF | Momencho | East | 27.34 | 88.81 |
| 3817 | TCF | Lachung | North * | 27.80 | 88.71 |
| 4086 | TCF | Yumthang | North* | 27.85 | 88.69 |
| 4282 | TCF | Yumthang | North* | 27.87 | 88.69 |
| 4649 | ALP | Yumesamdong | North* | 27.91 | 88.69 |
|  |  |  |  |  |  |

TrSDF- Tropical Semi-deciduous forests, TrMBF- Tropical moist and broad-leaved forests, TBF- Temperate broad-leaved forests, TCF- Temperate coniferous forests, SAP- Sub-alpine vegetation, AP- Alpine zone, FAS- Farm-based agroforestry system, MOAS- Mandarin orange-based agroforestry system, LCAS-Large cardamom-based agroforestry system.
