# Supplementary material for: Ecological niche modelling of two water-dependant birds informs the conservation needs of riverine ecosystems outside protected area network in the Eastern Himalaya, India
Source: PLoS One. 2023 Nov 9;18(11):e0294056. doi: 10.1371/journal.pone.0294056 (PMC10635460; doi:10.1371/journal.pone.0294056)
Supplement: Attachment — Submitted filename: Response to Reviewers comments.docx [file pone.0294056.s003.docx]

**Response to Reviewers Comments**

**PONE-D-23-07258**

Ecological niche modelling of two water-dependant birds informs conservation needs of riverine ecosystems outside protected area network in the Eastern Himalaya, India

**General Comment**: The manuscript entitled “Ecological niche modelling of two water-dependant birds informs conservation needs of riverine ecosystems outside protected area network in the Eastern Himalaya, India” is an interesting study but suffer from some major methodological flaws. Hence, I would recommend “Major Revision” at this stage. The authors should try to improve their manuscript as per the following suggestions:

**Comment 1**. As far as I understand, the authors have outlined “distance to water” to be the main factor for determining species presence of Blue Whistling Thrush and White-capped Water Redstart. The authors report that “distance to water” was calculated as Euclidean distance from the vector file of drainage systems of the DEM of the study area. If this is the case, then wouldn’t the resolution of DEM determine the output of “distance to water”? Also, the resolution of DEM and resolution of sampling effort does not match (If I am not wrong)? As per my understanding, “flow distance” is a better measure for measuring geographic distances in river networks. The reason being that Euclidean distance represents shortest straight line distance while flow distance joined by river flow. So, Euclidean distance might misrepresent the distance to river as it may indicate those sites that actually do not have any river water. The authors should consider this fact.

**Author response**: Yes, our results showed that ‘distance to water’ is more important for White-capped water redstart than Whistling thrush. We have revised the analyses based on reviewer comments. We have now used the data downloaded from HydroSHEDS which has high resolution data of global river works. This is a vector file representing the actual river flow. We created raster using euclidean distance tool in the same resolution (1x1 km) as our samples and pother predictor variables. This is a standard method for species distribution modeling approaches involving ‘distance to water’ layers. For example Mothes et al 2020 used the same method to model distribution of imperiled wood turtle (a water-dependant species) in the North America. We have cited this paper for the method we used in our analysis.

**Comment 2**. My second major concern is with respect to determining conservation needs (as reflected in the title) for bird species that themselves fall in the “Least Concern” Category of Red List (Blue Whistling Thrush and White-capped Water Redstart). The study area (Sikkim) is a hot-spot of avifaunal diversity with many endemic and threatened species. Why determine conservation priorities based on the most common species?

**Author response**: We agree with the reviewer in this context as conservation actions till now are mostly focused on rare and at-risk species. However, common species also have their own ecological and functional roles in ecosystems. Conservation of such species is critical to prevent them from rapid decline and local extinctions. Although these species are ‘least concern’ they might have specific ecological and habitat requirements which make them vulnerable at local scales. Also, we used these common species as indicator species of riverine ecosystems where they share space with many other sympatric species. The importance of addressing the conservation needs of common species are highlighted in Lindenmayer et al. 2011 (cited). We have made necessary changes in the revised manuscript now highlighting these issues

**Comment 3**. Another major flaw is that the conservation priorities have been determined based on just geographic and climatic factors. Wouldn’t it be more prudent to use “forest cover classes” as one of the predictor in SDM analyses as the study is with respect to birds?

**Author response**: Thank you for the suggestion. In the revised version we included land use and land cover layer (which also represents forest classes) as one of the predictor variables along with other predictor variables. However, this variable hasn’t turned out to be an important factor for the distribution of both species.

**Comment 4**. I am not sure if use of only one SDM technique (MaxEnt) is good for predicting habitat suitability for small datasets. It would be worthwhile to use “ensemble modelling approach” for determining habitat suitability. For example, see the work of Ahmad et al. (2020) who used ensemble modelling approach for predicting habitat suitability of Himalayan goral.

**Author Response**: Among many SDM approaches MaxEnt is reported to be one of the best method for predicting species with even small number of occurrence records (Pearson 2007).

[Pearson, R. G., Raxworthy, C. J., Nakamura, M., & Townsend Peterson, A. (2007). Predicting species distributions from small numbers of occurrence records: a test case using cryptic geckos in Madagascar. *Journal of biogeography*, *34*(1), 102-117.]

Although several new modeling approaches have been evolved in recent years, MaxEnt is still considered to be among the best ones due to its simplicity and high-performance. Kaky et al 2020 highlighted that single-algorithm modelling methods, particularly *MaxEnt*, are capable of producing distribution maps of comparable accuracy to *ensemble* methods.

Further there are studies showing no particular benefit to using ensembles over individual tuned models.

Examples:

[Kaky, E., Nolan, V., Alatawi, A., & Gilbert, F. (2020). A comparison between Ensemble and MaxEnt species distribution modelling approaches for conservation: A case study with Egyptian medicinal plants. *Ecological Informatics*, *60*, 101150.

Hao, T., Elith, J., Lahoz‐Monfort, J. J., & Guillera‐Arroita, G. (2020). Testing whether ensemble modelling is advantageous for maximising predictive performance of species distribution models. *Ecography*, *43*(4), 549-558.

**Comment 5**. The authors state that the protected area boundaries were sourced from World database of protected areas (WDPA). WDPA database does not include “Kitam Bird Sanctuary”. Was this PA included in the study or not? Also, Figure 3 depicts incorrect boundary of “Kyongnosla Alpine Wildlife Sanctuary”. Please cross-check. Also, name the individual PA in Figure 3.

**Author Response**: Thank you for highlighting this issue. We have included the Kitam Bird Sanctuary in the map using the boundary collected from Sikkim Forest Department. This was added to the WDPA data base that we created for the final PA area shape file. We cross-checked with the available data and the boundary of Kyongnosla Alpine Wildlife Sanctuary seems to be correct (<https://wiienvis.nic.in/WriteReadData/UserFiles/image/PAs_Map_Database/images/sikkim2.jpg>).

**Comment 6**. In Table 1, habitat suitability values for Elevation zone 6000-7000 m have been given for two bird species. Are these areas potential habitats for these bird species?

**Author Response**: After the revised analysis, there is only negligible area (less than 1 square km) above 6000m as suitable habitat (only for Blue Whistling Thrush). Although the model shows it as potential habitat, we will need further surveys to confirm their actual presence in these elevations. Considering the extreme weather conditions it is less likely that the species might actually occur there although our model shows some suitability.

**Comment 7**. Lines 98-106: The authors mention in detail the various kinds of anthropogenic pressures threatening the Sikkim Himalayan ecosystems? What is the use if no such anthropogenic threat is included in the analyses of suitable habitats?

**Author Response**: In conservation perspective, SDM analysis are mainly used for delineating most suitable habitats than identifying predictor variables. In modelling, we need to use samples and predictor layers in same resolution. Accounting multiple anthropogenic threats as a layer in SDM especially for such complex areas like Himalaya may not be practical. However, once we identify their potential habitats using other variables (such as bioclimatic) we can go further exploring most suitable habitats and understand the threats at local level. Our analysis will help to further investigate the threats in the riverine ecosystems of Sikkim Himalaya. Nonetheless we have highlighted these issues because they are very important from conservation point of view.

**Comment 8**. Would dam-building threaten the habitat of the two bird species (given the fact the Sikkim has one of the highest dam densities in the Himalaya and in the study the authors have concluded “distance to water” as the main predictor of species presence). Again, here I feel that if the authors had included some other bird species that ranks higher in IUCN red list, the study would had become very interesting.

**Author Response**: Dam-building is of course a threat to the riverine ecosystems in the region. As mentioned above (in response to comment 2), common species also need attention as they undertake specific ecological and functional roles in respective ecosystems. Moreover, our study species act as indicator species of riverine ecosystem and share habitats with many other important water-dependant species. Hence, our approach may help multiple species if we could develop strategies for these riverine habitats.
