# Supplementary material for: Ecological niche modelling of two water-dependant birds informs the conservation needs of riverine ecosystems outside protected area network in the Eastern Himalaya, India
Source: PLoS One. 2023 Nov 9;18(11):e0294056. doi: 10.1371/journal.pone.0294056 (PMC10635460; doi:10.1371/journal.pone.0294056)
Supplement: Attachment — Submitted filename: Response to Reviewers_R2.docx [file pone.0294056.s004.docx]

Response to Reviewers Comments

PONE-D-23-07258

Ecological niche modelling of two water-dependant birds informs conservation needs of riverine ecosystems outside protected area network in the Eastern Himalaya, India

Dear Editor,

We thank you for providing us a constructive review for our manuscript submitted to PLoS ONE. We have revised our manuscript based on reviewer’s comments and incorporated changes accordingly. There were two major concern of the reviewers (1) to change the model species from common birds to IUCN red listed species, and (2) to change modelling approach from MaxEnt to ensemble. I would like to mention that we have fully addressed concern 2 and reanalysed our model (ensemble) and addressed all the concerns raised by reviewer. Regarding the change of model bird species, we have provided strong justification below for why we cannot change the species from common to IUCN red listed. Almost all researchers focus only on IUCN red listed species but these common species are equally important from conservation point of view and are neglected by researchers and conservationists. Hence, we stick to the common species because our entire aim of this MS will be diluted if we change the model species . We believe that our manuscript is now in a better shape to consider for publication in your esteemed journal.

Review Comments to the Author

Reviewer #1: I have now completed the review of the revised manuscript entitled "Ecological niche modelling of two water-dependant birds informs conservation needs of riverine ecosystems outside protected area network in the Eastern Himalaya, India". While I find that the authors have addressed some of my comments from the previous review, I find that they have still not addressed my two major comments:

(i) They have still used only two most "common species" namely "Blue Whistling Thrush" and "White-capped Water Redstart" for determining conservation prioritization. In my previous review, I had highlighted the fact that conservation prioritization only works best when it is done for IUCN Red List species or Endemic species. I will strongly request the authors to redo SDM analyses by including IUCN Red List Species of Sikkim Himalaya and then do conservation prioritization exercise. Right now, the results hold no significance as the two subject species (Blue Whistling Thrush and White Capped Water Redstart) are prevalent everywhere in the study area irrespective of the threats. In a nutshell, they do not serve as good ecological indicators for threats. Hence, it would be best for the authors to gather species presence for the IUCN Red List species of Sikkim through primary/secondary databases and then perform conservation prioritization exercise.

We thank the reviewer for this comment but as we highlighted as part of the reply to first review comments, we would like to mention that our study is using these common species as indicators of riverine ecosystems. The main aim of this paper itself is to look for distribution and threats experienced by common species because most study focus only on IUCN listed species and these currently common species are always ignored, and not taken into consideration. Common species often play vital roles in ecosystem functions and processes. Globally, conservation strategies are mostly focused on threatened species and rarely explored the potential of using common species as indicators of critical ecosystems. Conserving common species is critical in retaining their key ecological and functional roles in ecosystems. However, conservation strategies are often focused only on threatened species and we need a holistic approach to preserve multiple ecosystem functions. For instance, a habitat conservation approach focusing on the riverine ecosystem could benefit multiple species including sympatric birds and other water-dependent taxa such as dragonflies, damselflies, fishes, amphibians and reptiles. Such approaches are needed to inform local and regional decision-makers on conservation or management options that anticipate the response of biodiversity to future climate and land use change (Please see the introduction part for rationale for using common species).

There are studies from the Himalayan region (cited in introduction) highlighting these water birds as cost-effective indicators of riverine habitats. Including more species especially threatened ones is challenging as we don’t have sufficient validated number of occurrences from the region. Additionally, the entire aim of the MS will change if we change the model species. Moreover, the region has not many IUCN threatened water-dependent species to include in the model. We used these common species to highlight their specialised niches although they are not threatened currently. However, we feel that such common species and other sympatric species might also have specific habitat requirements that needs to be preserved to protect them from future declines or local extinctions. We made modifications in the text also to highlight the importance of common species and also using them as indicators. We consider our study address multiple species in the ecosystem rather than just addressing conservation of these two target species. In this way we believe that the work is still relevant. Hence, we stick to the common species because our entire aim of this MS will be diluted if we change the model species.

(ii) The authors have still not used "ensemble modelling approach". I had highlighted in the previous review also that databases like Chelsa climate database that the authors have used are of global resolution. As such SDMs work best when local level climate models are used. I know that such databases do not exist for the Himalaya or Sikkim Himalayan region. Therefore, in these cases it is best to use ensemble modelling approach where climate simulations from different global databases are used and then the mean value is taken as a representation of the habitat/climate of the study area. This process removes any inherent bias with respect to downscaling of data from the global to the regional levels. Therefore, I will again suggest the authors to use different global climate model databases to perform SDM exercise and then take the mean values.

Thanks for your suggestion. We have now revised our analysis using ‘ensemble’ approach. We used five algorithms (combination of machine-learning and regression models) to generate combined output of the model. We hope that the model is now more robust.

Note: Ensemble approaches are recommended for avoiding the bias of using single algorithm but the same time there are studies highlighting that individually tuned models (like Maxent with customised feature settings) are very efficient in many cases where we have less number or spatially aggregated occurences as we highlighted in first revision.

Regarding Chelsa- studies show that the climate datasets from Chelsa is reported to be more efficient for modeling studies in mountain environments like the Himalaya (Reference: Maria B, Udo S. Why input matters: Selection of climate data sets for modelling the potential distribution of a treeline species in the Himalayan region. Ecol Model. 2017;359: 92–102. doi:10.1016/j.ecolmodel.2017.05.021).

We once again thank the editor and reviewer for providing valuable inputs to the MS which helped us to revise it significantly.
